# Vitamin D Deficiency and Rheumatoid Arthritis: Epidemiological, Immunological, Clinical and Therapeutic Aspects

**DOI:** 10.31138/mjr.30.2.94

**Published:** 2019-06-29

**Authors:** Behzad Heidari, Karimollah Hajian-Tilaki5, Mansour Babaei

**Affiliations:** 1 Mobility Impairment Research Center; Babol University of Medical Sciences, Babol, Islamic Republic of Iran; 2 Clinical Research Development Unit of Rouhani Hospital; Babol University of Medical Sciences, Babol, Islamic Republic of Iran; 3 Department of Internal Medicine, Division of Rheumatology; Babol University of Medical Sciences, Babol, Islamic Republic of Iran; 4 Department of Statistics and Epidemiology, School of Medicine; Babol University of Medical Sciences, Babol, Islamic Republic of Iran

**Keywords:** Disease activity, immunomodulation, rheumatoid arthritis, vitamin D deficiency, therapy

## Abstract

**Background/Aim:**

Vitamin D displays an immunologic effect which can modulate function of Th17-related cytokines and thereby prevent perpetuation of inflammation in chronic disorders like rheumatoid arthritis (RA). This review aims to conduct a literature review to provide a summary of recent studies addressing the relationship between vitamin D deficiency and RA based on epidemiological, immunological and therapeutic aspects.

**Methods:**

PubMed, Scopus and Google scholar were searched for relevant papers published between 2000–2018.

**Results:**

Low intake of vitamin D increases the risk of incident RA, and vitamin D deficiency has been shown to be inversely associated with RA activity in most of these studies. However, characteristics of RA and serum vitamin D status differ across the studies. The results of studies on the effect of supplemental vitamin D in RA vary, from no efficacy to significant improvement in disease activity, as well as quality of life. This should be attributed to variations in dosage of vitamin D, duration of treatment, baseline serum vitamin D in RA patients and characteristics of RA across diverse studies.

**Conclusion:**

Current data indicate a therapeutic potential for vitamin D in RA. However, further studies are needed to identify an optimal and effective dosage, duration of treatment and patients who will get the best benefit from the treatment.

## INTRODUCTION

Vitamin D deficiency is highly prevalent in the general population particularly in patients with musculoskeletal and autoimmune disorders.^1–3^ Distribution of vitamin D deficiency varies across various populations because of variations in diet, demographic features, ethnicity, geographic region, seasonality and severity of air pollution. Almost one-third of the US population have serum vitamin D levels less than 20 ng/ml.^3^ The results of a systematic review of 195 studies comprised of 168,000 individuals from 44 countries revealed vitamin D deficiency in 37.3% of the participants.^4^ Vitamin D deficiency is more prevalent in patients with autoimmune disorders, and in patients presenting in primary health centres in particular rheumatology clinics.^5–7^ Current observations have related vitamin D deficiency with many clinical and pathophysiological conditions, as well as with the development and exacerbation of several inflammatory or noninflammatory disabling musculoskeletal diseases including rheumatoid arthritis (RA).^8–11^ There has been a growing interest to elucidate the relationship between vitamin D and immunity, as well as the contribution of vitamin D deficiency in the development or exacerbation of autoimmune diseases particularly RA.^8,12,13^

Existing data on the association of vitamin D with severity of RA and the effect of vitamin D supplementation on RA are contradictory, and do not lead to a single conclusion. The present narrative review aims to conduct a literature review to elucidate this context by providing a summary of recent studies addressing the relationship between vitamin D deficiency and RA, based on epidemiological, immunological and therapeutic aspects. For this purpose, current studies in MEDLINE, Scopus, and Google Scholar were searched for relevant published English-language studies. In addition, the references of the selected papers were searched to find eligible studies between 2000 to 2018 by using the following key words: RA, vitamin D, supplementation, deficiency, disease activity, treatment.

## VITAMIN D DEFICIENCY AND IMMUNOPATHOGENESIS OF RA

Many observational studies have found decreased risk of certain types of cancers, cardiovascular disease, type 2 diabetes, and autoimmune diseases at serum 25-OHD levels greater than 28–32 ng/ml.^3^ At present, serum 25(OH)D concentrations <20 ng/ml and 21–29 ng/ml are considered deficient and insufficient levels respectively.^14^ Nonetheless, maintenance of serum 25(OH)D at levels of 40–60 ng/ml is suggested to be ideal and up to 100 ng/ ml is safe.^15^

The immune cells express vitamin D receptors (VDR) and produce 1,25(OH)2D, which is the biologically active metabolite of vitamin D3. This hormone is locally synthesized from conversion of 25(OH)D to 1,25(OH)2D by immune cells - particularly the dendritic cells and macrophages - within an autocrine/paracrine system.^2,3,8,13,16^ The tissue level of 1,25(OH)2D and its immunomodulatory activity is dependent on serum 25(OH)D concentration, as well as on cytokines that are produced by the immune cells. The level of 1,25(OH)2D in tissues is not dependent on parathyroid hormone, but is regulated by circulating serum 25(OH)D and by proinflammatory cytokines that are produced from the immune cells. Low level of serum 25(OH) D decreases the 1.25(OH)2D synthesis, and leads to decreased function of autocrine/paracrine system, whereas increasing circulating serum 25(OH)D concentration to sufficient level by vitamin D supplementation, improves the function of autocrine/paracrine system.^8,17^

The locally synthesized 1,25(OH)2D has a tissue-specific immunomodulating effect and control on the growth and functions of the immune cells.^18^ It inhibits Th1/Th2 differentiation and decreases self-reactive T-cells functions through downregulation of the antigen-presenting cells.^17^ The Th1, Th2, and Th17 cells play an important role in the coordination of the immune cells at the initiation of an inflammatory process. Vitamin D shifts the adaptive immune system from Th17 and Th1 cells towards Th2 cells and regulatory T cells.^17^ The presence of vitamin D deficiency impairs physiological activities in directing Th1 towards Th2-driven condition, and results in cytokine production towards Th17 cells. Since imbalance in Th1/ Th17 cells and hyperfunctioning of Th17 cells are both characteristics of RA, therefore, vitamin D may play a role in the pathogenesis of Th1-driven autoimmune diseases such as RA.^9^ Thus, vitamin D deficiency by increased production of cytokines may perpetuate inflammatory process to chronic inflammation.^17,19–21^

An inverse correlation between serum 25(OH)D concentration and Th17-related cytokines has been shown.^22,23^ Th17 proinflammatory cytokines stimulate osteoclastic differentiation and increase cartilage destruction and bone erosions, whereas 1, 25(OH)2D inhibits dendritic cell proliferation and maturation, promotes dendritic apoptosis and dendritic-dependent T-cells activation.^2,8^ Hence, vitamin D deficiency state as an environmental factor may put susceptible individuals at greater risk of autoimmune diseases through disturbing immunomodulatory effects.^24^ A systematic review of 219 cross-sectional and interventional studies on the association between vitamin D and autoimmune diseases revealed that hypovitaminosis D in genetically predisposed subjects can impair self-tolerance by disturbing immune cells regulation, and vitamin D supplementation has a potential role in the prevention of autoimmune diseases.^25^

## VITAMIN D INTAKE AND RA

Many studies have addressed the association between vitamin D intake and incident RA. Merlino et al. found an inverse association between vitamin D intake and RA in the Iowa Women’s Study. In this prospective longitudinal study, higher intake of vitamin D was associated with decreased risk of RA by 34%.^26^ This study has several limitations, including lack of data regarding sunlight exposure as well as measurements of serum 25(OH)D over the study period, and absence of clinical examination to confirm the diagnosis of clinical RA. A meta-analysis of 3 cohort studies comprised of 215,757 participants, indicated a 24.2% lower risk of incident RA in subjects in the highest group for total vitamin D intake as compared with those in the lowest group.^27^ Another systematic review and meta-analysis of 668 studies revealed that supplementation of vitamin D for 3 months reduced the prevalence of anti-dsDNA test positivity and RA recurrences.^28^ In contrast, many prospective longitudinal studies have found no association between dietary intake of vitamin D and the development of RA or systemic lupus erythematosus.^29–33^ In one study, the presence of serum vitamin D deficiency several years prior to diagnosis of RA was not associated with the disease onset, or seropositivity for rheumatoid factors, or anticyclic-citrullinated peptide antibodies.^31^ Another study found no increased risk of RA in vitamin D-deficient subjects.^32^ In a study of 2,162 Korean women aged 19 years and over, vitamin D status was not associated with the development of RA.^33^

The relationship between vitamin D intake and incident RA is difficult to be explained, because the main source of vitamin D is sunlight exposure and dietary vitamin D does not always correlate with serum 25(OH)D levels, because vitamin D intake constitutes a small proportion of body requirements. Furthermore, RA is a multifactorial disease where genetic and environmental factors are also contributed to its development. In addition, progression of RA from the preclinical stage to established disease may last several months to years, and thus, periodic measurements of vitamin D intake or serum 25(OH) D level are not reliable measures for estimating serum vitamin D status.

## ASSOCIATION OF SERUM VITAMIN D WITH RA ACTIVITY

Many studies have found a negative relationship between activity of RA and serum 25(OH)D concentration.^34^ The results of a longitudinal study of patients with RA revealed an association of hypovitaminosis D with disease activity in recent onset RA over 12 months follow-up study.^35^ Nevertheless, the results of studies on the association between serum 25(OH)D and disease activity (DA) in RA are conflicting (*Table 1*).^35–47^

**Table 1.** Studies on the association between serum vitamin D concentration and disease activity in rheumatoid arthritis.

**Authors**	**Study population (No)**	**Serum 25(OH)D status ng/ml**	**Results**	**Study design**
Kostoglou^38^	(44) RA vs (NA) controls	15.26+1.7 25.5+1.6	Negative correlation between vitamin D and DAS 28, CRP, and ESR	Case-control
Quintana-Duque^40^	(70) RA vs (70) controls	27.13+13.4 33.74+16.7 p= 0.01	Baseline 25(OH)D deficiency was associated with disease severity in early onset RA	Case-control
Vojinovic et al.^48^	(625) RA vs (276)controls	17 6+9.7 18.9+9.4 p= 0.0615	Negative correlation between vitamin D and DAS28-CRP	Cross-sectional study
Raczkiewicz et al.^43^	(97) RA vs (28) controls	Vitamin D deficiency 76.3% vs 78.6%), p=.75	Negative correlation between DAS28 and HAQ	Case-control
Rajaee et al.^53^	(93) new onset RA vs (31) controls	33.47+7.8 vs 30.03+2.3 p=.63	Negative correlation between vitamin D and DAS28	Case-control
Zakeri et al.^44^	(66) RA	30.5+28.9 ng/ml	Inverse association between vitamin D and DAS28-ESR, Number of tender joint, swollen joint, duration of morning stiffness	Cross-sectional
Di Franco et al.^35^	(37) RA	24.4+11.9 ng/ml	Negative association between vitamin D and DAS28, proportion of treatment response and remission	Longitudinal retrospective
Hong et al.^45^	(130)RA and (80) controls	43.1+15.6 vs 57.9+15.9 nmol/l p=0.01	Negative correlation between vitamin D and tender joint, swollen joints, joint pain, morning stiffness, and HAQ score as well as with IL-17, IL-23	Case-control
Abourazzak et al.^46^	(170) RA	Serum 25(OH)D < 30 ng/ml	Vitamin D deficiency was associated with severity of RA by OR=2.91(95%CI,1.31–6.44)	Cross-sectional
Haque et al.^51^	(62) RA	Serum 25(OH)D < 30 ng/ml in 61%	An inverse association of vitamin D deficiency with DAS28 and HAQ in active RA but not in remission	Cross-sectional
Moghimi et al.^39^	(87) active RA vs (71) silent RA	49.3+38.1 vs 64.6+43.6 mol/l p=0.022	Inverse association between serum vitamin D and RA activity	Case-control
Baker et al.^55^	(499) active RA	Serum 25(OH)D deficiency in 48%	No association between vitamin C deficiency and DAS28, inflammatory markers	Cross-sectional
Matsumoto et al.^54^	(181) RA vs (186) controls	Serum 25(OH) D was significantly lower in RA	No correlation between vitamin D deficiency and RA activity	Case-control
Pakchotanon et al.^50^	239 RA	28.79 ng/ml	No association with tender, swollen joint count, DAS28, HAQ score	Case-control
Haga et al.^56^	302 RA	Vitamin D deficiency 33.4%	No correlation between vitamin D and DAS28	Cross-sectional

RA; Rheumatoid arthritis; CRP: Creactive protein; ESR: Erythrocyte sedimentation rate; NA:Not available; DAS28: Disease activity score on 28 joints; OR: Odds ratio; HAQ: Health assessment questionnaire

In one longitudinal study by Patel et al., there was an independent negative correlation between serum 25(OH) D and DAS28. The value of DAS28 decreased proportionately by increasing serum 25(OH)D concentration over the study period.^36^ In another large study comprised of 1,191 patients with RA, a negative correlation between serum 25(OH)D and DA was observed only in non-supplemented RA patients.^42^ Similarly, a negative correlation between serum vitamin D and DAS28-CRP was observed in a multicentre study of 625 RA patients with mean age of 55±11 years, mean serum 25(OH)D of 17.62±9.76 ng/ml and mean disease duration of 11±9 years.^48^ A negative association of vitamin D with DAS28 has also been reported in other studies including two meta-analyses.^35,37,44–47^

A recent umbrella review summarized the health outcomes that have been associated with vitamin D concentrations including 107 systematic reviews, 74 meta-analyses of observational studies, and 87 meta-analyses of randomized controlled trials. Regarding the role of vitamin D supplementation, the umbrella review showed a definite association of vitamin D with RA activity.^49^

Nonetheless, a number of studies have failed to show a significant association between serum 25(OH)D and disease activity in RA.^50–56^

Matsumoto et al. found no correlation between serum 25(OH)D and diseas activity in a study of 181 RA patients and 186 matched controls.^54^ Similarly, Baker et al. in a study of 499 active RA patients found no association between vitamin D deficiency and DAS28, as well as no association between baseline vitamin D deficiency and change in DAS28.^55^ Another study of 239 patients with mean serum 25(OH)D level of 28.79 ng/ml, there was no correlation between serum 25(OH)D and DAS28.^43^

Contradictory results on the association of vitamin D and disease activity in RA can be attributed to a possible contribution of several factors such as sunlight exposure, physical activity, severity of RA, dietary vitamin D, prevalence of vitamin D deficiency in the general population, and treatment of RA. These factors may differently affect the results across studies.

On the other hand, the presence of low serum 25(OH)D in RA may be a consequence of inflammation, because vitamin D in itself is an acute phase protein and may decrease during an acute inflammatory process.^57^ As a result, in patients with active RA, low vitamin D may be a parameter of inflammation rather than the cause of disease activity. Therefore, the inflammatory process may have a confounding effect on the association between serum 25(OH)D and disease activity in RA.

## THE BENEFICIAL EFFECT OF VITAMIN D SUPPLEMENTATION IN RA

Treatment of RA aims to relieve pain and suppress the inflammatory process.^58^ The origin of pain in RA may be inflammation, structural damages, or alteration in central pain processing.^59^ The pain in itself is an important clinical feature and a measure for assessment of RA activity. An association between vitamin D deficiency and musculoskeletal pain has been shown, which is responsive to vitamin D supplementation.^60,61^ The critical role of Th17-related cytokines in the pathogenesis of RA and their inverse relation with vitamin D indicates a therapeutic potential for vitamin D supplementation in RA.^22,23^ Vitamin D decreases production of Th17-related cytokines,^23,24^ and so vitamin D supplementation in RA patients might provide not only an immunomodulatory effect, but also can exert additional benefits in favour of muscle strength, falls and osteoporosis.^20,45,61,62^

Several randomized clinical trials which have been conducted to evaluate the effect of vitamin D in RA found improvement in pain, disease activity and general health (*Table 2*). A randomized double-blind placebo-controlled study of 80 patients with musculoskeletal diseases revealed that addition of 4000 IU cholecalciferol to the analgesic regimen for 3 months provided a greater decrease in pain. Furthermore, the serum TNFα level decreased by 54.3% and the PGE2 level decreased by 39.2% as compared to placebo group.^63^

**Table 2.** Clinical trials using vitamin D supplementation in the treatment of rheumatoid arthritis.

**Authors (year of study)**	**Study design**	**Baseline treatment+ vitamin D dosages**	**Study population**	**Outcome measure**	**Results**	**Duration of study**
Gendelman et al.^63^ (2015)	RCT	Oral cholecalciferol 4000IU/d vs placebo	patients with rheumatic diseases	Changes in pain and inflammatory cytokines	Significant decline in pain and TNFa, PGE2, and leukotriene B compared with placebo	3 months
Buondonno et al.^64^ (2017)	RCT	MTX+GC + single dose of 300.0000 IU cholecalciferol vs MTX+GC alone	21 patients 19 RA controls	Changes in global health	Addition of vitamin D was significantly more effective in ameliorating global health	3 months
Lourdudoss et al.^70^ (2008)	Prospective	Dietary Intake of vitamin D, omega 3 and folate +Standard therapy with MTX with or without GC	727 early RA	EULAR response	Higher intake of vitamin D was associated with increased EULAR response by OR=1.80 (1.14–2.83)	3 months
Andjelkovic et al.^68^ (1999)	Open-label study	Alfacalcidiol 2 μg/day + DMARDs	19 RA	Changes in DA	Evaluation of disease activity showed complete remission in 9, satisfactory response in 8 and no effect in 2 patients	3 months
Chandrashekara et al.^69^ (2017)	Open-label interventional study	Vitamin D at 60.000 IU weekly for 6 weeks, 60.000 IU monthly up to 3 months + DMARDs	150 vitamin D deficient RA mean dd=78 months	Changes in DAS 28	Significant improvement in DAS28 compared with baseline	3 months
Salesi et al.^65^ (2012)	RCT	Addition of 50.000 IU vitamin D to MTX for 12 weeks vs placebo	Active RA	Improvement in DAS 28 score	No significant difference in vitamin D vs placebo 44% vs 33.4%	12 weeks
Dehghan et al.^67^ (2013)	RCT	Addition of 50.000 IU vitamin D weekly vs placebo to baseline DEMARDs for 6 months	80 RA on remission with serum vit D< 30 ng/ml	DAS28 and proportion of disease recurrence at endpoint	No significant in proportion of RA recurrences in vitamin D and placebo groups	6 months
Yang^66^	Randomized controlled open-label	Addition of alfacalcidol 0.25 μg, twice a day to baseline DMARDs vs Baseline drugs without alfacalcidol	192 Vitamin D deficient RA at remission	DAS28 and disease recurrences in patients with and without vitamin D	Recurrence rate in subgroups with and without vitamin D treatment did not differ	24 months

RCT: Randomized clinical trial; RA: Rheumatoid arthritis; DA: Disease activity; DAS28: Disease activity score on 28 joints; DMARDs: Disease modifying anti-rheumatic drugs; MTX: Low dose methotrexate; GC: Glucocorticoid; dd: Disease duration.

In another randomized double-blind placebo-controlled study of early RA, addition of a single dose of 300.000 IU cholecalciferol to the standard treatment ameliorated patient’s overall health. In this study, mean serum 25(OH)D level increased from 16+4 ng/ml at baseline to 28+4.3 ng/ml at endpoint.^64^ Nonetheless, another double-blind placebo-controlled study of active RA showed that, addition of 50.000 IU cholecalciferol for 12 weeks to baseline methotrexate did not improve DAS28 in spite of increasing serum 25(OH)D from 107+28.1 to 125+22.4 nmol/L in the treatment group. In this study, baseline serum 25(OH)D concentrations in both treatment and control groups were at sufficient levels.^65^

The effect of vitamin D on the recurrence of RA has been investigated in two clinical trials. Yang et al. examined the effect of vitamin D supplementation for the prevention of RA recurrences in a study of 377 patients with RA who achieved remission. The patients were divided into vitamin D sufficient and deficient groups. The patients in the vitamin D-deficient group were classified as vitamin D treatment and no treatment subgroups. After two years of follow-up, the recurrence rate in the sufficient group was significantly lower than in the non-vitamin D treatment deficient subgroup (29.5% vs 16.7% respectively). However, treatment of vitamin D deficiency, in spite of raising serum 25(OH)D from 20.6+9.1 to 25.5+8.9 ng/ ml, did not affect the rate of recurrences as compared to non-treated subgroup (19% vs 16.7%).^66^

Similarly, another double-blind placebo-controlled study of RA patients at remission with serum 25(OH)D <30 ng/ ml for two months, 50.000 IU weekly vitamin D supplementation for 6 months failed to prevent disease recurrences as compared to placebo.^67^

The effect of a single high dose of vitamin D, or weekly doses of 50.000 IU or 60.000 IU for 3 months has been evaluated in a few observational studies without a control group. In a small open-label study of patients with severe and moderate RA activity, addition of high-dose oral calcidiol for 3 months to the standard DMARDs therapy provided higher remission rate and greater efficacy on disease activity.^68^

Similarly, Chandrashekara et al. in a study of vitamin D deficient active RA, added 60.000 IU vitamin D weekly for 6 weeks, followed by 60.000 IU monthly to the baseline DMARDs therapy, for a total duration of 3 months. At endpoint, DAS28 decreased significantly as compared to baseline value. In this study, the serum 25(OH)D levels increased from 10.05±5.18 at baseline to 57.21±24.77 ng/mL at endpoint.^69^ Chandrashekara et al. in another study of patients with early RA, found that higher intake of dietary vitamin D prior to the initiation of DMARDs resulted in better outcome.^70^

As shown in *Table 2*, the results of supplemental vitamin D in RA vary across diverse studies, since these studies differ with respect to design, patient characteristics in particular baseline serum 25(OH)D, dosages and duration of vitamin D therapy, and outcome measures. Nevertheless, the results of observational studies are prone to bias; therefore, their results are not as reliable as randomized clinical trials. A systematic review and meta-analyses of 76 case-control and 19 observational or interventional studies which assessed the effect of vitamin D therapy on various autoimmune diseases revealed a potential role for vitamin D in autoimmune disease prevention; although, evidence of benefit for vitamin D supplementation in randomized clinical trials is lacking.^71^

## CONCLUSION

The immunomodulating effect of vitamin D against Th17-related cytokines, provided an interest to investigate the association of serum vitamin D with the incidence and activity of RA as well as the effect of vitamin D supplementation in RA. Data in relation to vitamin D intake and RA are conflicting because influence of sunlight exposure as a main source of vitamin D has been ignored in most studies. Similarly, studies that assessed the association between vitamin D and RA activity are heterogenous in respect to baseline serum vitamin D levels from severe deficiency state to sufficient levels, physical activity, dietary vitamin D. Moreover, in studies which examined the effect of vitamin D in RA, dosages of vitamin D and severity of disease, duration of treatment and the outcome measures vary across different studies. Current data indicates a potential role for vitamin D in the treatment of RA. Nevertheless, it is unclear, which dose of vitamin D provides more benefit, or how much serum 25(OH)D level should be increased to be effective?

Treatment of RA aims to suppress the inflammatory process particularly at the early disease stage when structural injuries are minimal. The presence of structural changes decreases response to anti-inflammatory drugs. Hypothetically, vitamin D-deficient RA patients are expected to have a better treatment response at earlier stages of RA; thus, raising serum 25(OH)D to higher level is expected to be more effective. Future studies should be directed towards efficacy of vitamin D on Th17-related cytokines in patients with early RA. Correlation between clinical responses and changes in these cytokines can be considered as a guide for evaluation of response to treatment. A causal relationship between vitamin D deficiency and RA requires a longitudinal study of healthy subjects with periodic assessment of vitamin D intake, serum 25(OH)D level and intermittent clinical examination to detect development of clinical RA.
